# Prevalence and antimicrobial resistance characterization of multidrug-resistant *Staphylococcus epidermidis* isolated from raw milk of dairy cattle and ewes

**DOI:** 10.1371/journal.pone.0334516

**Published:** 2025-11-12

**Authors:** Inamullah, Mustafa Kamal, Farhad Badshah, Shehryar Khan, Ghassan Tayh, Mourad Ben Said, Eliana Ibáñez-Arancibia, Patricio R. De los Ríos Escalante, Huma Fatima, Muhammad Ikram, Hanène Belkahia, Tahir Usman

**Affiliations:** 1 College of Veterinary Sciences and Animal Husbandry, Abdul Wali Khan University Mardan, Mardan, Pakistan; 2 Department of Zoology, Abdul Wali Khan University Mardan, Mardan, Pakistan; 3 State Key Laboratory of Animal Biotech Breeding, Institute of Animal Science, Chinese Academy of Agricultural Science, Beijing, China; 4 Shenzhen Branch, Guangdong Laboratory of Lingnan Modern Agriculture, Key Laboratory of Livestock and Poultry Multi-Omics of MARA, Agricultural Genomics Institute at Shenzhen, Chinese Academy of Agricultural Sciences, Shenzhen, China; 5 Department of Biotechnology, Abdul Wali Khan University Mardan, Mardan, Pakistan; 6 Laboratory of Microbiology, National School of Veterinary Medicine of Sidi Thabet, University of Manouba, Sidi Thabet, Tunisia; 7 Department of Basic Sciences, Higher Institute of Biotechnology of Sidi Thabet, University of Manouba, Sidi Thabet, Tunisia; 8 PhD Program in Sciences mentioning Applied Molecular and Cell Biology, La Frontera University, Temuco, Chile; 9 Laboratory of Engineering, Biotechnology and Applied Biochemistry—LIBBA, Department of Chemical Engineering, Faculty of Engineering and Science, La Frontera University, Temuco, Chile; 10 Department of Biological and Chemical Sciences, Faculty of Natural Resources, Catholic University of Temuco, Temuco, Chile; 11 Nucleus of Environmental Sciences, Faculty of Natural Resources, Catholic University of Temuco, Temuco, Chile; 12 Department of Zoology, Women University Mardan, Mardan, Pakistan; 13 Department of Pharmacy, Abdul Wali Khan University Mardan, Mardan, Pakistan; The Rockefeller University, UNITED STATES OF AMERICA

## Abstract

Milk is a vital and widely consumed food, but contamination by biological, chemical, and physical factors can lead to milk-borne diseases. Mastitis, particularly subclinical mastitis (SCM), is a significant biological factor that deteriorates milk quality. Among the 135 agents causing SCM, *Staphylococcus epidermidis*, a Gram-positive coagulase-negative staphylococcus, plays a notable role. The excessive and indiscriminate use of antibiotics in treating mastitis has led to the emergence of multidrug-resistant (MDR) strains, posing threats to both animal and human health. This study aimed to assess the prevalence of SCM and identify MDR *S. epidermidis* isolates in raw milk samples from dairy cattle and ewes, and explore the presence of antibiotic resistance genes (*mecA*, *tetK*, and *ermC*) in these isolates. A total of 310 milk samples were collected from Holstein Friesian and Cholistani cattle, as well as ewes under transhumant and sedentary husbandry systems. The results revealed a 26% overall prevalence of SCM, with a higher incidence in ewes (31.34%) than in cattle (21.87%). Within cattle, SCM prevalence rate was 40% in the Cholistani breed and 17.69% in the Holstein breed. *S. epidermidis* was detected in 12.9% of the samples, with 72.5% of these isolated from SCM cases. Antibiotic susceptibility tests showed high resistance rates to penicillin and erythromycin (95%), moderate resistance rates to cotrimoxazole, doxycycline, clindamycin, and chloramphenicol, and low resistance rates to levofloxacin and ciprofloxacin (5%). Notably, 50% of the isolates were MDR. Among the resistance genes, *ermC* was most prevalent (87.5%), followed by *tetK* (80%) and *mecA* (45%). These findings underscore the widespread presence of *S. epidermidis* in both healthy and SCM-affected dairy animals, as defined by elevated somatic cell counts, highlighting its dual role as a commensal organism and a potential pathogen, resulting in significant implications for antibiotic resistance management in dairy farming.

## Introduction

Milk plays a vital role in the production of dairy products in Pakistan, which is the third largest milk-producing country in the world [1]. Although milk is a highly nutritious food, its production can be hindered by several factors, including poor management, hygiene issues, genetic abnormalities, malnutrition, reproductive problems, and diseases such as mastitis [2]. Mastitis, an inflammatory disease of the mammary gland, is primarily caused by bacteria like *Escherichia coli*, *Staphylococcus* spp., and *Streptococcus* spp.*,* and sometimes by fungi. This condition typically occurs during the dry-off and early lactation periods in dairy herds, leading to alterations in milk quality and abnormal gland appearance [3]. The clinical symptoms of mastitis include discomfort, elevated body temperature, flu-like symptoms, and redness, tenderness, warmth, and swelling in the affected area [4].

Mastitis in dairy animals can manifest as either clinical cases with visible symptoms or as subclinical infections, where no visible or microscopic signs are apparent [5]. Subclinical mastitis (SCM) is particularly concerning as it often goes undetected, resulting in a higher somatic cell count due to bacterial infections, negatively impacting milk yield and composition, and leading to financial losses from reduced milk production and discarded milk [6,7].

Among the bacterial pathogens responsible for intra-mammary infections (IMI), *Staphylococcus* spp. are the most commonly identified in small ruminants [8]. Notably, *S. epidermidis*, a coagulase-negative staphylococcus (CoNS), is a predominant cause of subclinical mastitis in dairy ruminants [9]. This opportunistic pathogen is characterized by its ability to form biofilms, a key virulence factor, and by its resistance to multiple antibiotics [10]. The genetic factors associated with biofilm formation and antibiotic resistance in *S. epidermidis* contribute significantly to its role as a causative agent of subclinical mastitis [11].

The rise of antibiotic-resistant strains of *S. epidermidis* poses a significant challenge in treating infections. Methicillin-resistant *Staphylococcus epidermidis* (MRSE) strains, in particular, are increasingly recognized as major pathogens due to their resistance to multiple antibiotics, leading to multidrug resistance (MDR) and limited treatment options [12,13]. The misuse and overuse of antibiotics in veterinary care, agriculture, and human medicine exacerbate the emergence of antibiotic-resistant bacteria [14,15]. In particular, the indiscriminate use of antibiotics in a population can contribute to the development of resistance, making it crucial to use antibiotics judiciously [16].

*S. epidermidis* is known for its extensive antimicrobial resistance and its ability to facilitate horizontal gene transfer, spreading resistance genes among staphylococcal populations [17]. This bacterium often harbors various resistance genes, including those responsible for methicillin resistance (*mecA*), tetracycline resistance (*tetK*, *tetL*, *tetM*, and *tetO*), and erythromycin resistance (*ermA* and *ermB*), among others [18,19]. These mechanisms of resistance, such as efflux pumps and ribosomal protection, complicate the treatment of infections caused by *S. epidermidis* [20]. In a recent study, Haq et al. [21] reported a prevalence of 30.32% for *Staphylococcus* in bovine milk samples collected from various regions in Pakistan. Similarly, Saeed et al. [22] identified *S. epidermidis* in milk samples of lactating women in Pakistan, while Talebi et al. [23] reported 25.24% prevalence rate of *S. epidermidis* in milk samples collected from cattle in Iran.

Given the public health implications of antibiotic-resistant *S. epidermidis* in dairy production, it is essential to monitor the prevalence and resistance profiles of this pathogen in milk. This study aimed to examine the prevalence of *S. epidermidis* in raw milk samples from lactating cattle and ewes, assess its antibiotic resistance profile, and identify the presence of key antibiotic resistance genes (*mecA*, *tetK*, and *ermC*).

## Materials and methods

### Ethical statement

This study was approved by the Advanced Studies and Research Board (ASRB) (Dir/A&R/AWKUM/2023/10014) of the Faculty of Chemical and Life Sciences, Abdul Wali Khan University Mardan, Pakistan.

### Study design

This study involved the collection of 310 milk samples, including 160 from cattle and 150 from ewes. Among the cattle, 130 samples were collected from Holstein Friesian breed and 30 from Cholistani breed. Cattle samples were obtained from various dairy farms in Punjab, Pakistan, using random sampling to ensure a representative population. Ewes’ samples were collected from flocks in the northern Khyber Pakhtunkhwa (KPK) province. All samples were immediately preserved in sterile falcon tubes containing 0.01 mg/mL potassium dichromate to prevent bacterial overgrowth and degradation during transport. The samples were kept at 4°C in an icebox and transported to the Genetics Laboratory of the College of Veterinary Sciences and Animal Husbandry (CVS&AH), Abdul Wali Khan University, Mardan, for further analysis.

### Diagnosis of subclinical mastitis

Subclinical mastitis in the milk samples was diagnosed by measuring the somatic cell count (SCC) using direct microscopy, following established protocols [24]. The SCC was determined using an automated cell counter calibrated according to the manufacturer’s instructions. Cattle were classified as healthy if the SCC was < 200,000 cells/mL, and as subclinical mastitis (SCM) cases if the SCC was > 200,000 cells/mL. For ewes, the classification thresholds were <400,000 cells/mL for healthy and >400,000 cells/mL for SCM cases, in accordance with the standards provided by Esteban-Blanco et al. [25].

### Confirmation post-isolation of *S. epidermidis*

To confirm the presence of *S. epidermidis*, milk samples were streaked onto Mannitol Salt Agar (MSA) plates and incubated at 37°C for 16–18 hours. One putative bacterial colony was picked from the MSA agar culture and sub-cultured onto fresh MSA plates. Biochemical tests were performed on purified colonies, including Gram staining, catalase test, and coagulase test, following the protocol described by Kivaria et al. [26]. Colonies that were Gram-positive, catalase-positive, and coagulase-negative were taken as *S. epidermidis* for molecular confirmation using species-specific *rdr* gene.

### Stock preparation

Pure cultures of *S. epidermidis* were inoculated into Luria-Bertani (LB) broth (Sigma-Aldrich, Merck, Germany) according to the manufacturer’s instructions. The inoculated broth was incubated at 37°C for 24 hours with continuous shaking. Growth was indicated by turbidity in the broth. For long-term storage, 500 µL of the bacterial culture was mixed with 1000 µL of 70% glycerol in sterile Eppendorf tubes and stored at −40°C for future use.

### DNA extraction

Genomic DNA was extracted from the biochemically confirmed *S. epidermidis* isolates using Chelex® 100 resin (Bio-Rad Inc., USA) as described by Lubna et al. [27], with minor modifications. A 5% Chelex solution was prepared, and 70 µL of this solution was mixed with a few fresh colonies of *S. epidermidis* in a sterile Eppendorf tube. The mixture was incubated at 45°C for 30 minutes, followed by centrifugation at 13,000 rpm for 5 minutes. The supernatant containing DNA was carefully transferred to a new Eppendorf tube. The presence and quality of DNA were verified using 1% agarose gel electrophoresis (Bio-Rad Inc., USA).

### Molecular identification of *Staphylococcus epidermidis*

The bacterial isolates identified by biochemical tests were confirmed by the molecular identification of *S. epidermidis*. Polymerase Chain Reaction (PCR) was employed to amplify the species-specific *rdr* gene (130 bp) using specific primers (Forward: 5’-AAGAGCGTGGAAAAAGTATCAAG-3’ and Reverse: 5’-TCGATACCATCAAAAAGTTGG-3’) [28]. A final reaction volume of 20 µL was used in a DNA thermal cycle (kyratec, Australia), the reaction mixture contained 10 µL of 2X Master Mix (Bio Basic Inc., Canada), 6 µL of PCR-grade water, 2 µL of extracted DNA, and 1 µL each of forward and reverse primers in 0.4 µM. The PCR conditions were as follows: initial denaturation at 94°C for 5 minutes, followed by 30 cycles of denaturation at 94°C for 30 seconds, annealing at 61.8°C for 30 seconds, and extension at 72°C for 45 seconds, with a final extension at 72°C for 10 minutes. The PCR products were analyzed by electrophoresis at 100 volts with a current of 60 mA for 30 minutes on a 2% agarose gel, stained with ethidium bromide, and visualized under a UV transilluminator. A 100 bp DNA ladder (Gene Ruler, Thermo Scientific, Lithuania) was used to determine the size of the amplified fragments.

### Antibiotic Susceptibility Test (AST)

The susceptibility of all isolates to commonly used veterinary antibiotics was assessed by the disc diffusion method using Mueller-Hinton agar plates. Eight antibiotics from seven different antibiotic classes were tested (Table 1). After a 24-hour incubation at 37°C, the diameter of the inhibition zones around each antibiotic disc was measured in millimetres. The results were interpreted according to the Clinical and Laboratory Standards Institute (CLSI) guidelines (2020). The presence of multidrug-resistant (MDR) strains was determined following the criteria defined by Magiorakos et al. [29]. If an isolate shows resistance to one or more agents from three or more antimicrobial classes, it is classified as multidrug resistant (MDR).

**Table 1:** Antibiotics tested for susceptibility of *Staphylococcus epidermidis* isolates.

Antibiotic class	Antibiotic disc	Code	Quantity (µg)
**Penicillinase-labile Penicillin**	Penicillin	P	10
**Macrolides**	Erythromycin	E	15
**Folate pathway antagonists**	Cotrimoxazole	COT	25
**Tetracyclines**	Doxycycline	DO	30
**Lincosamides**	Clindamycin	CD	2
**Phenicoles**	Chloramphenicol	C	30
**Fluoroquinolones**	Ciprofloxacin	CIP	5
	Levofloxacin	LEV	5

### Prevalence of antibiotic resistance genes

To screen for antibiotic resistance genes, PCR assays were performed using specific primers targeting for the *mecA*, *tetK*, and *ermC* genes (Table 2). The reaction mixture for each gene contained 10 µL of 2X Master Mix (Bio Basic Inc., Canada), 6 µL of PCR-grade water, 2 µL of DNA template, and 1 µL each of forward and reverse primers in 0.4 µM. The PCR conditions for *mecA* and *tetK* genes included an initial denaturation at 95°C for 10 minutes, followed by 30 cycles of denaturation at 95°C for 30 seconds, annealing at 56.6°C for *tetK* and 60.2°C for *mecA* for 30 seconds each, and extension at 72°C for 30 seconds. For the *ermC* gene, the initial denaturation was performed at 94°C for 5 minutes, followed by 30 cycles of denaturation at 94°C for 1 minute, annealing at 55°C for 1 minute, and extension at 72°C for 1 minute. Final extensions were performed at 72°C for 10 minutes for *mecA* and *tetK*, and 7 minutes for *ermC*. The amplicons were analyzed on a 2% agarose gel using electrophoresis at 100 volts with a current of 60 mA for 30 minutes, stained, and visualized as described above.

**Table 2:** PCR primers used for detection of antibiotic resistance genes.

Gene	Nucleotide sequence (5’-3’)	AT (°C)	Base pairs (bp)	Reference
*mecA*	F: AAAATCGATGGTAAAGGTTGGC	60.2	532	[30]
	R: AGTTCTGCAGTACCGGATTTGC			
*tetK*	F: GTAGCGACAATAGGTAATAGT	56.6	360	[31]
	R: GTAGTGACAATAAACCTCCTA			
*ermC*	F:AATCGTCAATTCCTGCATGT	55	299	[32]
	R: TAATCGTGGAATACGGGTTTG			

**Abbreviations:** AT: Annealing temperature.

## Results

### Prevalence of subclinical mastitis (SCM)

In this study, 310 milk samples were analyzed for somatic cell counts to determine the prevalence of subclinical mastitis (SCM). The overall prevalence was 26% (82/310). Among the samples, 160 were collected from cattle, and 150 from ewes. The prevalence of SCM was slightly higher in ewes at 31.34% (47/150) compared to 21.87% (35/160) in cattle. Among cattle breeds, the prevalence of SCM in Cholistani breed was 40% (12/30), and in Holstein Friesian breed the prevalence was 17.69% (23/130). In ewes, the prevalence was similar across husbandry systems, with 32.18% (28/87) in the transhumant system and 30.15% (19/63) in the sedentary system (Table 3).

**Table 3:** Prevalence of subclinical mastitis (SCM) in cattle and ewes by breed and husbandry system.

Livestock type	Category	Prevalence of SCM
**Cattle**	All	21.87%
	Cholistani	40.00%
	Holstein Friesian	17.69%
**Ewes**	All	31.34%
	Transhumant	32.18%
	Sedentary	30.15%

### Prevalence of *Staphylococcus epidermidis*

All 310 milk samples were examined for the presence of *S. epidermidis* by streaking on Mannitol Salt Agar (MSA). Positive isolates were characterized by identify colonies with typical *S. epidermidis* morphology (small, white, or greyish), coagulase-negative, gram-positive cocci and catalase-positive and molecularly verified through the presence of *rdr* gene. Out of 310 samples, 12.90% (n = 40) tested positive for *S. epidermidis*. Of these 40 isolates, 27.5% (n = 11) were from healthy samples, while 72.5% (n = 29) were from SCM-positive samples.

### Factors influencing the prevalence of *Staphylococcus epidermidis*

The prevalence of *S. epidermidis* was analyzed across different factors, including species, breed, and husbandry system. The prevalence was almost similar between cattle and ewes, at 13.12% (21/160) and 12.67% (19/150), respectively. In cattle, *S. epidermidis* was isolated from 33.33% (7/21) of healthy samples and 66.67% (14/21) of SCM-positive samples. In ewes, 21.05% (4/19) of isolates were from healthy samples, while 78.95% (15/19) were from SCM-positive samples. The *p*-value between species was non-significant. Among cattle breeds, the prevalence was higher in the Cholistani breed (23.34%, 7/30) compared to the Holstein Friesian breed (10.76%, 14/130). In the Cholistani breed, 28.57% of isolates were from healthy samples, while 71.43% were from SCM-positive samples. The prevalence difference between breeds of cattle was non-significant, *p* > 0.05. For ewes, the prevalence of *S. epidermidis* was higher in the transhumant system (63.15%, 12/19) compared to the sedentary system (36.85%, n = 7/19). In the transhumant system, 75% of isolates were from SCM-positive samples, while in the sedentary system, 85.71% of isolates were from SCM-positive samples. The prevalence difference between transhumant and sedentary pastoralism was non-significant *p* > 0.05 (Table 4).

**Table 4:** Distribution of prevalence of *Staphylococcus epidermidis* on the basis of healthy and subclinical mastitis (SCM) cases by species, breed, and husbandry system.

Livestock type	Category	Total Positive	Healthy Positive	SCM Positive
**Cattle**	All	13.12%	33.33%	66.67%
	Cholistani	23.34%	28.57%	71.43%
	Holstein Friesian	10.76%	35.71%	64.29%
**Ewes**	All	12.67%	21.05%	78.95%
	Transhumant	63.15%	25%	75%
	Sedentary	36.85%	14.29%	85.71%

### Antibiotic Susceptibility Test (AST)

The *S. epidermidis* isolates were subjected to antibiotic susceptibility testing (AST) using the disc diffusion method, following CLSI 2020 guidelines. Highest resistance rates were observed to penicillin and Erythromycin (95%), followed by cotrimoxazole (27.5%), doxycycline (25%), clindamycin (17.5%), and chloramphenicol (15%). Low resistance rates were observed to levofloxacin and ciprofloxacin (5%). Based on the AST results, the isolates were categorized into three groups: susceptible, intermediate, and resistant (Table 5).

**Table 5:** Antibiotic sensitivity pattern of *Staphylococcus epidermidis* isolates (n = 40).

Antibiotics	Resistant (%)	Intermediate (%)	Susceptible (%)
**Penicillin**	38 (95%)	0 (0%)	2 (5%)
**Erythromycin**	38 (95%)	0 (0%)	2 (5%)
**Cotrimoxazole**	11 (27.5%)	0 (0%)	29 (72.5%)
**Doxycycline**	10 (25%)	0 (0%)	30 (75%)
**Clindamycin**	7 (17.5%)	16 (40%)	17 (42.5%)
**Chloramphenicol**	6 (15%)	0 (0%)	34 (85%)
**Ciprofloxacin**	2 (5%)	2 (5%)	36 (90%)
**Levofloxacin**	2 (5%)	0 (0%)	38 (95%)

### Detection of multidrug-resistant *Staphylococcus epidermidis*

**Fig 1:**
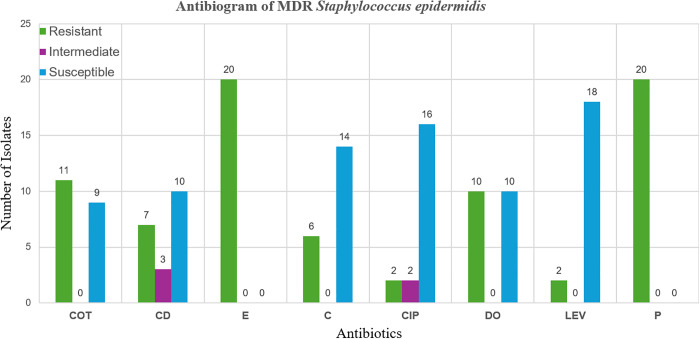
Antibiogram of multidrug-resistant *Staphylococcus epidermidis* isolated from raw milk samples.

Among the isolates, 50% (n = 20/40) were classified as multidrug-resistant (MDR), showing resistance to three or more classes of antibiotics. Out of MDR strains 45% (n = 9/20) were isolated from ewes while 55% (n = 11/20) were isolated from cattle. In ewes 44.45% (n = 4/9) were isolated from healthy samples, while 55.55% (n = 5/9) were isolated from SCM samples. Similarly, 36.36% (n = 4/11) of the MDR isolates were from healthy samples of cattle, while 63.63% (n = 7/11) were isolated from SCM milk samples of cattle. The MDR isolates exhibited the highest resistance rates to erythromycin and penicillin, with additional resistance to clindamycin, chloramphenicol, and doxycycline. The lowest resistance rates were observed to levofloxacin and ciprofloxacin (Fig 1).

### Screening for antibiotic-resistant genes

PCR screening for antibiotic-resistant genes was conducted on all *S. epidermidis* isolates. The analysis revealed that 87.5% (35/40) of isolates carried the *ermC* gene, 80% (32/40) carried the *tetK* gene, and 45% (18/40) carried the *mecA* gene. Table 6 shows that ten strains isolated from ewes are multi-gene resistant, harboring the *mec*A, *tet*K, and *erm*C genes. In contrast, strains from cattle carried only one or two of these resistance genes. Most strains 95% (18/19) isolated from ewes harboured *mecA* gene which is absent in isolates from cattle.

**Table 6:** Characteristics of the 40 *S. epidermidis* isolates recovered from raw milk of dairy cattle and ewes.

Strain ID	Animal species	Antibiotics	Resistance genes detected
S1	Ewes	E	*tet*K, *erm*C
S2*	Ewes	P, E, COT	*mec*A, *tet*K, *erm*C
S3	Ewes	P, E	*mec*A, *tet*K, *erm*C
S4	Ewes	P, E	*mec*A, *tet*K, *erm*C
S5	Ewes	P, E	*mec*A, *tet*K, *erm*C
S6	Ewes	P, E	*mec*A, *tet*K,
S7*	Ewes	P, E, COT, CD, LEV	*mec*A, *tet*K, *erm*C
S8	Ewes	P, E	*mec*A, *tet*K, *erm*C
S9*	Ewes	P, E, DO	*mec*A, *erm*C
S10*	Ewes	P, E, DO	*mec*A, *erm*C
S11	Ewes	P, E	*mec*A, *tet*K, *erm*C
S12	Ewes	P, E	*mec*A, *erm*C
S13*	Ewes	P, E, DO, C	*mec*A, *erm*C
S14*	Ewes	P, E, COT, DO, CD, CIP,	*mec*A, *tet*K, *erm*C
S15	Ewes	P, E	*mec*A, *tet*K,
S16*	Ewes	P, E, DO, CD	*mec*A, *erm*C
S17*	Ewes	P, E, DO	*mec*A, *erm*C
S18	Ewes	P, E	*mec*A, *tet*K, *erm*C
S19*	Ewes	P, E, CD, C	*mec*A, *tet*K, *erm*C
C1*	Cattle	P, E, COT, CD, LEV	*tet*K, *erm*C
C2	Cattle	P, E	*tet*K, *erm*C
C3	Cattle	P	*tet*K,
C4	Cattle	P, E	*tet*K, *erm*C
C5*	Cattle	P, E, DO	*erm*C
C6	Cattle	P, E	*tet*K, *erm*C
C7*	Cattle	P, E,COT	*tet*K, *erm*C
C8*	Cattle	P, E, COT	*tet*K,
C9*	Cattle	P, E, COT	*tet*K, *erm*C
C10	Cattle	P, E	*tet*K, *erm*C
C11*	Cattle	P, E, COT	*tet*K, *erm*C
C12	Cattle	P, E	*tet*K, *erm*C
C13*	Cattle	P, E, COT, C	*tet*K, *erm*C
C14	Cattle	P, E	*tet*K
C15*	Cattle	P, E, COT, DO, CD	*tet*K, *erm*C
C16	Cattle	P	*tet*K, *erm*C
C17*	Cattle	P, E, COT	*tet*K, *erm*C
C18	Cattle	P, E	*erm*C
C19*	Cattle	P, E, COT, DO, C	*tet*K, *erm*C
C20	Cattle	E	*tet*K, *erm*C
C21*	Cattle	P, E, DO, C, CIP	*tet*K, *erm*C

* = Multi drug resistant, P (Penicillin), E (Erythromycin), COT (Cotrimoxazole), DO (Doxycycline), CD (Clindamycin), C (Chloramphenicol), LEV (Levofloxacin), CIP (Ciprofloxacin).

## Discussion

This primary objective of the study was isolating and identifying *Staphylococcus epidermidis* in raw milk sample of cattle and ewes. *S. epidermidis* is known to be a food-borne pathogen in ruminants and it may cause subclinical mastitis and milk quality degradation. While, in humans it is a widespread commensal on the skin and mucosal lining and is not a classical food-borne pathogen. However, the concern with its presence in raw or unpasteurized milk is the possible health impact of high bacterial burden, and specifically the possibility of horizontal spread of antimicrobial resistance (AMR) genes to human microbiota, including pathogens like *Staphylococcus aureus*. Our study found SCM prevalence of 26%, which aligns with various global reports, such as 28.9% in New South Wales [33], 38% in Pakistan [34], while higher than the reported prevalence 15.2% in Serbia by Zutic et al. [35]. However, the SCM prevalence recorded in our study is lower than the prevalence 86.2% reported in Uganda [36]. The variation in SCM rates can be attributed to factors such as environmental conditions, management practices, pathogen diversity, breed, species, age, milk yield, and biosecurity measures. For instance, different breeds and species exhibit varying resistance to mastitis, and factors such as climate and hygiene practices play a significant role in infection rates [26].

In our study, SCM prevalence was 40% in Cholistani cattle and 17.69% in Holstein Friesian This contrasts with findings from other regions where Holstein Friesian cattle were more prone to SCM than native breeds [37,38]. The discrepancies might be due to differences in farm hygiene, climate, or sample size. Ewes had a higher SCM prevalence (31.34%) compared to cattle (21.87%). This contrasts with other studies where SCM was more common in cattle [39]. The difference may be due to variations in mammary gland anatomy, management practices, or immune responses. Ewes’ higher SCM prevalence could be attributed to shorter teat canals [40]. Regarding husbandry systems, our study found similar SCM rates in both transhumant (32.18%) and sedentary (30.15%) systems. This is inconsistent with other reports that indicate higher SCM prevalence in transhumant systems compared to sedentary ones [41,42]. The similar rates observed in our study could be due to uniform environmental conditions in the sampling region.

We identified *S. epidermidis* in 12.90% of milk samples. Our results aligns with findings from various regions; like Sumathi et al. [43] reported 16% prevalence in India, while Piessens et al. [44] recorded 11.9% in Belgium. However, our recorded prevalence of *S. epidermidis* is lower than those recorded in other studies, such as 25.24% in Iran, 37% in Pakistan, and 70.07% in Spain [11,23,27]. Similarly, our recorded prevalence was slightly higher than 7.5% reported in Belgium [45]. The variability in *S. epidermidis* prevalence is influenced by pathogen distribution, environmental conditions, genetic diversity, hygiene practices, and milking procedures [46].

In the present study, 27.5% of *S. epidermidis* isolates were obtained from milk samples of dairy animals that tested negative for subclinical mastitis (SCM), indicating the potential presence of *S. epidermidis* in animals without infection. Comparatively, Rall et al. [47] reported that *S. epidermidis* was isolated from 17.5% of milk samples from healthy cows, while 15.4% of isolates were obtained from cows suffering from mastitis, while Altuntaş [48] observed a significantly higher prevalence, detecting *S. epidermidis* in 55% of milk samples from healthy mothers. Ensuring standardized practices from milk collection to sale and providing specialized training to personnel can minimize contamination risks and safeguard consumer health [49]. Staphylococcal mastitis can be effectively managed with antimicrobial therapy; β-lactams, tetracyclines, and macrolides are commonly used as therapeutic alternatives. However, the increasing frequency of drug-resistant strains has weakened the effectiveness of these treatments.

Our study also assessed antibiotic resistance patterns of *S. epidermidis*. We observed high resistance rates to penicillin and erythromycin (95%), with lower resistance rates to levofloxacin and ciprofloxacin (5%). These findings are consistent with previous studies [50–53]. Resistance to penicillin is widespread due to its extensive use in veterinary and medical treatments, driven by mechanisms such as β-lactamase production and altered penicillin-binding proteins [54]. The observed resistance patterns underscore the need for continued research and action to address antibiotic resistance as a critical public health issue [55].

For gram-positive microbes, erythromycin is an antibiotic that can be used instead of cephalosporin, penicillin, and other beta-lactams. It has been used for a long time to treat a variety of infections [56]. High prevalence of erythromycin-resistance *Staphylococcus* in bovine mastitis isolates was recently reported in Pakistan (53.1%) [57] and Colombia (50%) [58]. In a Chinese study, high resistance rates were reported in *S. aureus* and coagulase-negative staphylococci strains to penicillin, followed by erythromycin and tetracycline. Meanwhile, the isolates exhibited low resistance rates to gentamicin, ciprofloxacin, and chloramphenicol [59]. In a Colombian study, *S. epidermidis* exhibited a significantly higher antibiotic resistance compared to other *Staphylococcus* species, suggesting that it has acquired virulence and resistance genes over time, enhancing its pathogenic potential [58].

We identified multidrug-resistant (MDR) strains in 50% of *S. epidermidis* isolates, aligning with findings from Nisar et al. [60] reported a similar MDR rate of 52.9%. However, other studies reported lower MDR rates in China and Brazil [61,62] and higher rates in Sudan and United States [63,64]. MDR in *S. epidermidis* can arise from inherent resistance mechanisms, beta-lactamase production, and biofilm formation, with uncontrolled antibiotic use contributing to resistance [16,65,66]. The presence of resistance factors, often conveyed through plasmids, highlights the complex nature of MDR patterns in *S. epidermidis* [67]. In Belgium, *S. epidermidis* had the highest number of multidrug-resistant (MDR) isolates among workers and animals on dairy, meat, and poultry farms, highlighting its potential role as a reservoir of antibiotic resistance in these ecosystems [68].

The widespread use of the antibiotics to treat mastitis, as well as other diseases or reproductive problems, may explain the high incidence of phenotypic resistance. Increased exposure to antibiotics can lead to the emergence of resistance strains, thereby contributing to the variability observed in the resistance profiles of the isolates [59]. In the present study, the genes conferring resistance to erythromycin and tetracycline, along with methicillin, were identified. Furthermore, our findings underscore the critical importance of including cefoxitin as a primary agents for screening methicillin resistance in staphylococci. Our investigation of antibiotic resistance genes revealed high prevalence of *ermC* (87.5%) and *tetK* (80%), with *mecA* showing a lower prevalence (45%). These results are consistent with previous studies, which reported similar or varying prevalence rates for these genes [69–71]. Staphylococci’s resistance to antibiotics is mostly associated with various resistance determinants, including the β-lactam resistance gene *mecA*, the tetracycline resistance genes *tet*, and the macrolide resistance genes *erm*. The presence of these resistance determinants and the high level of resistance to commonly used veterinary antimicrobials may explain the persistence of staphylococci in dairy herds [59]. The horizontal gene transfer among coagulase-negative staphylococci facilitates the spread of resistance genes [18,72]. In order to ensure the effective use of antibiotics and lower the risk of resistance development and transmission, it is imperative to monitor antimicrobial resistance.

Our study screened for methicillin resistance by detecting the *mecA* gene, which was confirmed in *S. epidermidis* isolates. Methicillin-resistant coagulase-negative staphylococci (MR-CNS) are increasingly recognized as significant threats in both human and animal health. Reports indicate their presence in cattle, animal handlers, and various environments [73], as well as in clinical and food samples [74]. Furthermore, MR-CNS have been identified in clinical isolates from hospitals [75]. Notably, 95% of *S. epidermidis* isolates from ewes carried the *mecA* gene, with the coexistence of *mecA*, *tetK*, and *ermC* resistance genes also observed. These findings highlight the concern that CNS may act as potential donors of the *mecA* gene to more pathogenic staphylococci, such as *S. aureus*. This situation poses a significant risk on ewe farms, with the potential for these strains to spread to other animals, humans, and the environment.

## Conclusion

This study underscores the significant prevalence of subclinical mastitis (SCM) in both cattle and ewes, with a notably higher incidence observed in ewes. The detection of *S. epidermidis* in both healthy and SCM-affected milk samples highlights its potential role in one-health dynamics, indicating that this pathogen could impact both animal health and food safety. The alarming rates of antibiotic resistance observed, particularly a striking 95% resistance level to penicillin and erythromycin, underscore the urgent need for improved antibiotic stewardship. In contrast, levofloxacin and ciprofloxacin exhibited comparatively lower resistance rates. Additionally, the identification of key antimicrobial resistance genes, especially *ermC* and *tetK*, and the high prevalence of multidrug-resistant strains, further accentuate the pressing need for effective surveillance and management strategies. Our study strongly recommending the use of cefoxitin for screening methicillin resistance in staphylococci. Addressing these challenges requires a multifaceted approach to improve milk quality, safeguard food safety, and reduce the economic and health impacts of mastitis. Implementing rigorous monitoring, enhancing hygiene practices, and promoting responsible antibiotic use are crucial steps in combating the rise of multidrug-resistant strains and ensuring the well-being of dairy livestock.

## Supporting information

S1 Table Data set. (XLSX)
