# Correction: Prevalence and antimicrobial resistance characterization of multidrug-resistant *Staphylococcus epidermidis* isolated from raw milk of dairy cattle and ewes

**DOI:** 10.1371/journal.pone.0356733

**Published:** 2026-08-20

**Authors:** Inamullah, Mustafa Kamal, Farhad Badshah, Shehryar Khan, Ghassan Tayh, Mourad Ben Said, Eliana Ibáñez-Arancibia, Patricio R. De los Ríos Escalante, Huma Fatima, Muhammad Ikram, Hanène Belkahia, Tahir Usman

There are errors in the author affiliations. The correct affiliations are as follows:

**1** College of Veterinary Sciences and Animal Husbandry, Abdul Wali Khan University Mardan, Mardan, Pakistan, **2** Department of Zoology, Abdul Wali Khan University Mardan, Mardan, Pakistan, **3** State Key Laboratory of Animal Biotech Breeding, Institute of Animal Science, Chinese Academy of Agricultural Science, Beijing, China, **4** Shenzhen Branch, Guangdong Laboratory of Lingnan Modern Agriculture, Key Laboratory of Livestock and Poultry Multi-Omics of MARA, Agricultural Genomics Institute at Shenzhen, Chinese Academy of Agricultural Sciences, Shenzhen, China, **5** Department of Biotechnology, Abdul Wali Khan University Mardan, Mardan, Pakistan, **6** Laboratory of Microbiology, National School of Veterinary Medicine of Sidi Thabet, University of Manouba, Sidi Thabet, Tunisia, **7** Department of Basic Sciences, Higher Institute of Biotechnology of Sidi Thabet, University of Manouba, Sidi Thabet, Tunisia, **8** PhD Program in Sciences mentioning Applied Molecular and Cell Biology, La Frontera University, Temuco, Chile, **9** Laboratory of Engineering, Biotechnology and Applied Biochemistry—LIBBA, Department of Chemical Engineering, Faculty of Engineering and Science, La Frontera University, Temuco, Chile, **10** Departamento de Ciencias Biológicas y Químicas, Facultad de Recursos Naturales, Universidad Católica deTemuco, Chile, **11** Núcleo de Estudios Ambientales, Facultad de Recursos Naturales, Universidad Católica de Temuco, Chile, **12** Department of Zoology, Women University Mardan, Mardan, Pakistan, **13** Department of Pharmacy, Abdul Wali Khan University Mardan, Mardan, Pakistan.

## References

[1] Inamullah, Kamal M, Badshah F, Khan S, Tayh G, Ben Said M, et al. Prevalence and antimicrobial resistance characterization of multidrug-resistant *Staphylococcus epidermidis* isolated from raw milk of dairy cattle and ewes. PLoS One. 2025;20(11):e0334516. doi: 10.1371/journal.pone.0334516 41223199 PMC12611168

